# Nanostructured copper/porous silicon hybrid systems as efficient sound-emitting devices

**DOI:** 10.1186/1556-276X-9-487

**Published:** 2014-09-11

**Authors:** Gonzalo Recio-Sánchez, Kyoko Namura, Motofumi Suzuki, Raúl J Martín-Palma

**Affiliations:** 1 Departamento de Ciencias Matemáticas y Físicas, Facultad de Ingeniería, Universidad Católica de Temuco, 4813302 Temuco, Chile; 2 Department of Micro-Engineering, Kyoto University, 615-8540 Kyoto, Japan; 3 Departamento de Física Aplicada, Universidad Autónoma de Madrid, Cantoblanco 28049, Madrid, Spain

**Keywords:** Porous silicon, Copper, Hybrid system, Nanostructure, Thermoplasmonics

## Abstract

In the present work, the photo-acoustic emission from nanostructured copper/porous silicon hybrid systems was studied. Copper nanoparticles were grown by photo-assisted electroless deposition on crystalline silicon and nanostructured porous silicon (nanoPS). Both the optical and photo-acoustic responses from these systems were determined. The experimental results show a remarkable increase in the photo-acoustic intensity when copper nanoparticles are incorporated to the porous structure. The results thus suggest that the Cu/nanoPS hybrid systems are suitable candidates for several applications in the field of thermoplasmonics, including the development of sound-emitting devices of great efficiency.

## Background

Nowadays, in a wide range of research fields including photonics, optoelectronics, engineering, and biomedicine, there is an increasing demand for multifunctional and affordable materials. In this regard, nanostructured porous silicon (nanoPS) has been demonstrated to be a good candidate for the development of multifunctional materials [1-3]. In addition to its tunable morphological and physicochemical properties, which greatly depend on the fabrication parameters, the relative simplicity and low cost of nanoPS processing make nanoPS a promising material for its use in many fields, photonics among them. Moreover, the compatibility of Si with standard electrochemical processes allows the fabrication of integrated metal/semiconductor hybrid systems [4].

In the particular case of sound-emitting devices, nanoPS has been demonstrated as a versatile material to fabricate efficient devices [5]. Since its optical and thermal properties can be easily tuned by changing its porosity, nanoPS offers great versatility aiming at adjusting its photo-acoustic response [6]. In addition, devices based on this material have been proposed as sound transmitters [7] or even to obtain three-dimensional images [8]. Furthermore, the photo-acoustic emission from noble metal nanoparticles is a property which can be exploited for different applications in several fields such as biomedicine [9], energy [10], microfluids [11], sound emitters [12], etc., since they are able to act as localized heat sources. This effect is based on the plasmon resonance of noble metal nanoparticles and can be optimized by adjusting their size and shape or by combining them with dielectric materials [13].

In the present work, the incorporation of Cu nanoparticles into nanoPS structures by photo-assisted electroless deposition is studied in detail. This experimental method represents an efficient and low-cost technique for the fabrication of metal/semiconductor hybrid systems. The photo-acoustic response of the resulting hybrid systems is analyzed, and the results show that these systems can be good candidates for the development of efficient sound-emitting devices.

## Methods

Porous silicon layers were fabricated by electrochemical etching of boron-doped p^+^-type silicon wafers (resistivity 0.01 to 0.05 Ω · cm; orientation <100>). The composition of the solution was 1:2 hydrofluoric (HF) (48 wt.%):ethanol (98 wt.%). The wafers were galvanostatically etched under illumination from a 100-W halogen lamp. The applied etching density current was 80 mA · cm^-2^ for 20 s to grow layers around 75% porosity and 750 nm of thickness.

The deposition of Cu nanoparticles over p^+^-type crystalline Si and nanoPS layers was carried out by immersing the substrates in CuSO_4_ (50 mM) and H_2_SO_4_ (1 mM) aqueous solutions at room temperature during different times. H_2_SO_4_ is used to stabilize the pH. The deposition was activated by turning on the 100-W halogen lamp during the appropriate time. Once the deposition is finished, the samples are washed and rinsed twice in water and twice in ethanol and subsequently dried with N_2_.

Field emission scanning electron microscopy (FESEM) images were acquired using a XL 30S FEG SEM (Philips, Amsterdam, The Netherlands). Fourier transform infrared spectroscopy (FTIR) was carried out using a Bruker IF-S66v spectrophotometer (Bruker AXS, Inc., Madison, WI, USA). Optical characterization was carried out using a Jasco V-560 UV-vis spectrophotometer (Halifax, Canada) equipped with a Hamamatsu R928 photomultiplier (Hamamatsu Photonics, Iwata, Japan). All measurements were taken in the wavelength range between 350 and 850 nm with a 1-nm interval and with 1-s integration time.

The photo-acoustic measurements were performed using a photo-acoustic cell (10 mm in diameter and 3.5 mm in height, MTEC Model 300, MTEC Photoacoustics, Inc., Ames, IA, USA). The samples were irradiated with a 785-nm wavelength laser whose intensity was modulated sinusoidally between 2 and 40 mW. The photo-acoustic signal was measured by means of a lock-in amplifier and recorded as a function of the laser modulation frequency over the range 1 to 100 kHz.

## Results and discussion

Succinctly, the deposition of Cu consists in the photoreduction of metal ions present in the solution, thanks to the reductive action of the semiconductor substrate, following a displacement reaction that is summarized as follows [14]:

(1) $$\text{Si}+2H_{2}O={\text{SiO}}_{2}+4H^{+}+4e^{-}$$

(2) $${\text{SiH}}_{x}+2H_{2}O={\text{SiO}}_{2}+\left(4+x\right)H^{+}+\left(4+x\right)e^{-}$$

(3) $${\text{Cu}}^{+2}+2e^{-}=\;\text{Cu}$$

**Figure 1:**
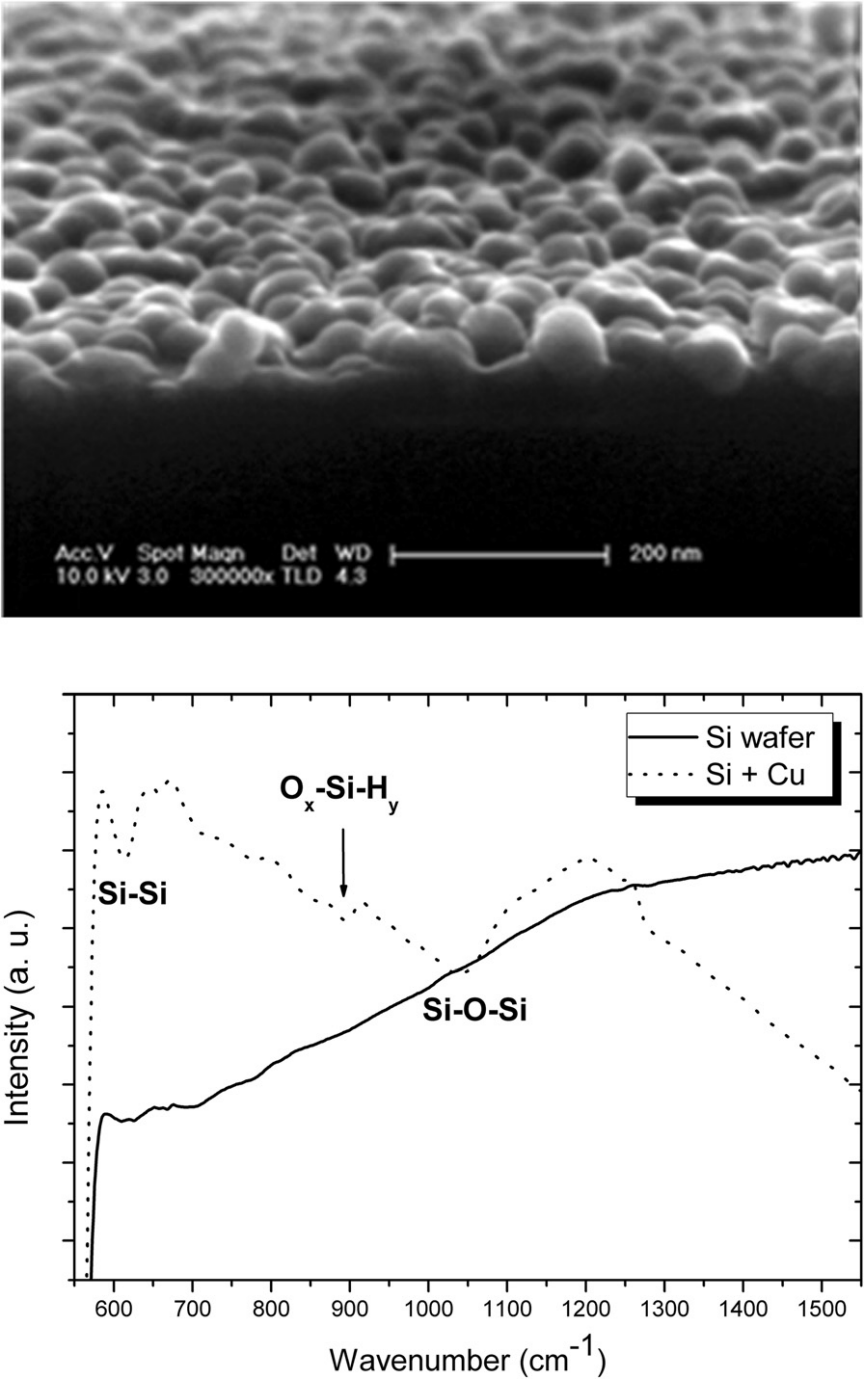
**FESEM image of Cu deposition and FTIR spectra.** (Top) FESEM image of Cu deposition on p^+^-type crystalline silicon. (Bottom) FTIR spectra of crystalline Si wafer before (Si wafer) and after (Si + Cu) Cu deposition.

In the first step of the displacement reaction, the Si atoms are oxidized meanwhile the water molecule is dissociated. This reaction is catalyzed by light given that photons create electron-hole pairs in the p-doped Si wafer. These electrons are available to react in the coupled redox reaction in which Si is oxidized and Cu is reduced. Due to the high reduction potential of Cu^2+^ with respect to the oxidation potential of Si, this reaction is thermodynamically favorable.After the immersion of the Si wafer into the copper solution for 320 s under illumination of the 100-W halogen lamp, an almost continuous Cu thin film is grown by cluster coalescence, which can be observed with the naked eye as a reddish film. Figure 1 (top) shows a typical FESEM image of a copper deposit on the surface of the Si wafer. It is observed that the morphology of the film is formed by interconnected copper nanoparticles. Additionally, the Cu nanoparticles show an average diameter of around 50 nm, and they form a thin film of 100 nm of thickness.

**Figure 2:**
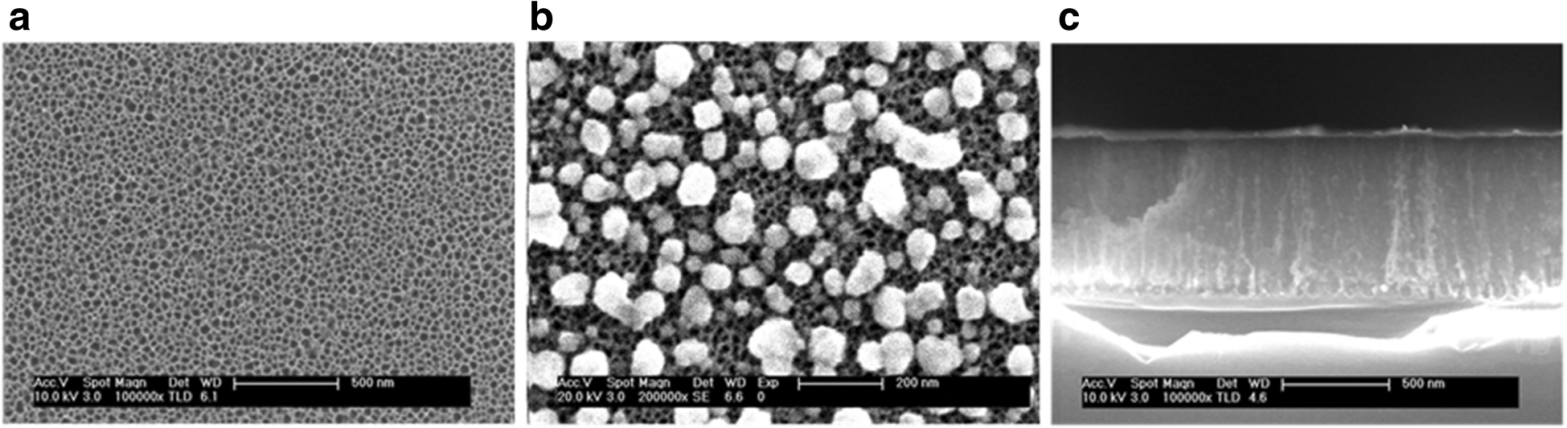
**FESEM images. (a)** Fresh nanoPS surface before immersion in the Cu solution, **(b)** after immersion in the Cu solution, and **(c)** cross section after immersion in the solution.

In order to verify that the Cu deposition is initialized by the oxidation of silicon, FTIR spectra were acquired before and after immersion of Si wafers in the copper solution (Figure 1 (bottom)). In the FTIR spectrum after the Cu deposition (Si + Cu), a wide band centered at 1,080 cm^-1^ associated to the presence of Si-O-Si bonds appears. Also, a contribution of surface O_*x*_-Si-H_*y*_ bonds appears at 885 cm^-1^, and the absorption of Si-Si bonds at 615 cm^-1^ is more intense, most likely due to the oxidation making their vibration easier.The surface of a typical fresh nanoPS layer is shown in Figure 2a. A porous structure with an average pore size around 40 nm is clearly observed. After the immersion of the nanoPS layer in copper solutions under illumination for 60 s, copper nanoparticles are nucleated on the surface as depicted in Figure 2b. From Figure 2b, it is noted that Cu nanoparticles have a pseudo-spherical shape and a wide size distribution, with a maximum around 100 nm of diameter. Figure 2c shows the cross section of a nanoPS layer after Cu electroless deposition. This figure shows a typical longitudinal porous structure and that the thickness of nanoPS layer is around 750 nm. In addition, some copper nanoparticles can be observed inside the porous structure, thus showing that Cu deposition is carried out not only over the PS surface but also inside the pores.

**Figure 3:**
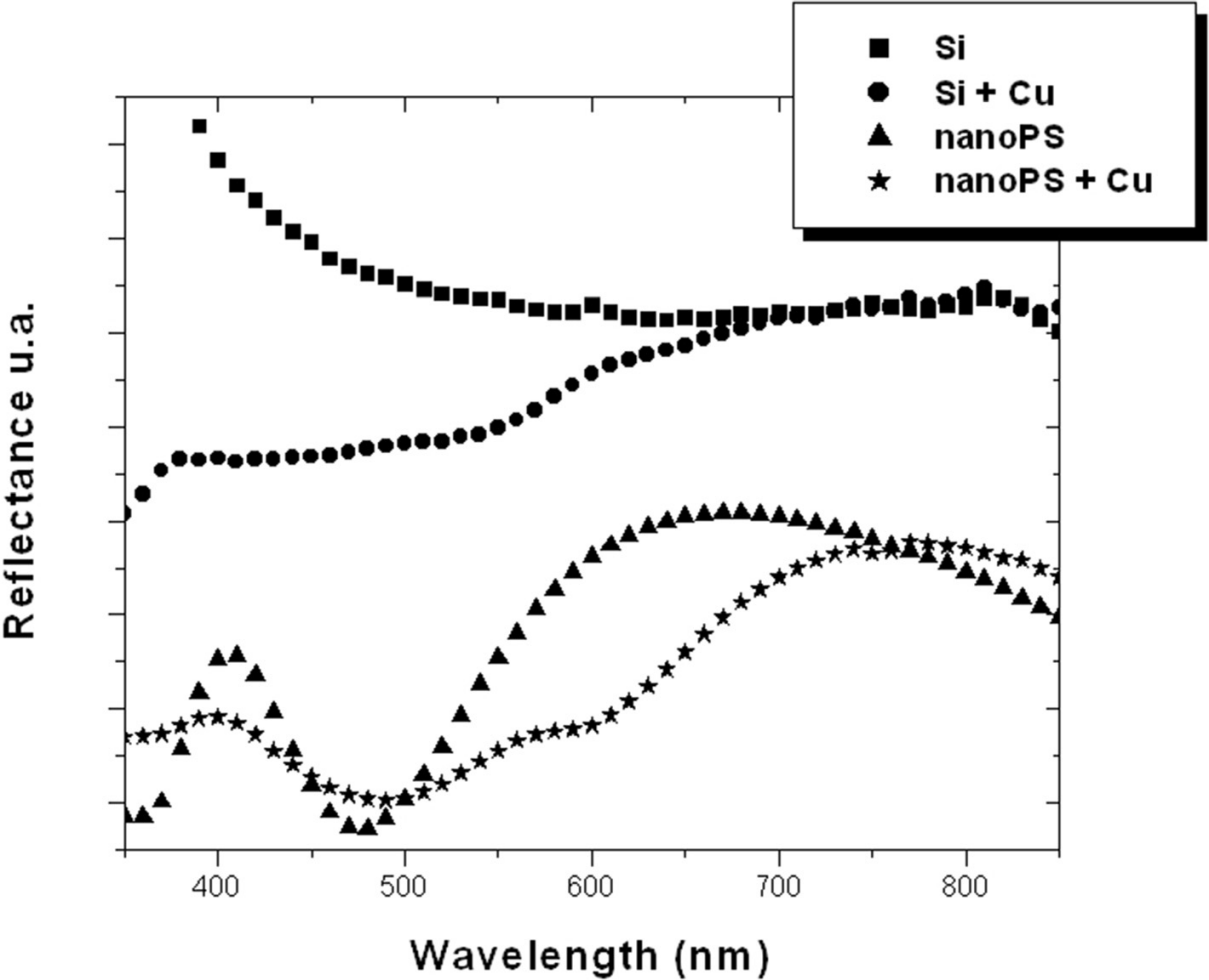
**Reflectance spectra.** Reflectance spectra of the Si wafer before (Si) and after (Si + Cu) Cu deposition and the nanoPS layer before (nanoPS) and after (nanoPS + Cu) immersion in Cu solution.

Given that the photo-acoustic response starts by the absorption of electromagnetic radiation, the optical response of these devices is a key parameter. As such, the optical behavior of the different structures was measured. The characteristic reflectance spectra are shown in Figure 3, from which it can be observed that after Cu deposition on c-Si, strong absorption from the metallic films is observed. For larger wavelengths (around 700 nm), the shape of the reflectance spectrum is quite similar to that of crystalline Si. However, for shorter wavelengths, notable absorption from the metallic films is appreciated, thus reducing the overall reflectance. The different absorption peaks are attributed to interband transitions of novel metals, where electrons from *d* orbitals are excited to the Fermi level [15]. In the case of copper, these transitions start at around 2.1 eV [16].

**Figure 4:**
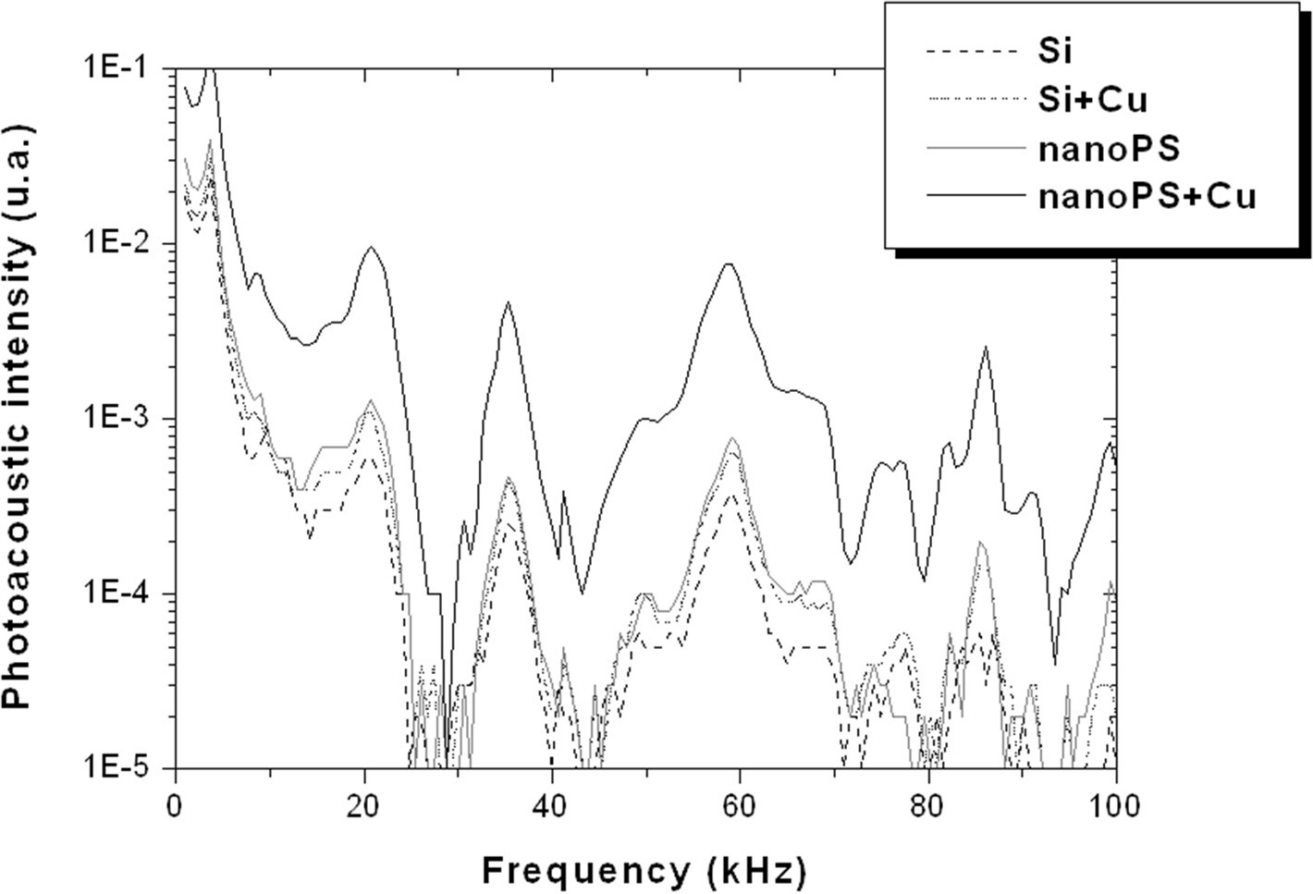
**Photo-acoustic spectra.** Photo-acoustic spectra of the Si wafer (Si), Si wafer after immersion in the Cu solution (Si + Cu), nanoPS layer (nanoPS), and nanoPS layer after Cu nanoparticle deposition (nanoPS + Cu). The photo-acoustic measurements were performed with a 785-nm wavelength laser.

Once nanoPS layers are formed on top of the c-Si substrate, the total reflectance is reduced due to light confinement processes inside the porous structure [17]. In the case of the nanoPS spectrum, typical interference fringes given by the thin film are clearly observed. After Cu nanoparticle deposition, an absorption shoulder attributed to the formation of Cu nanoparticles appears between 550 and 650 nm. This absorption shoulder can be understood as a combination of surface plasmon resonance form Cu nanoparticles and absorption corresponding to intraband electronic transitions [18]. The wide absorption band can be associated to the wide range of sizes and shapes of the Cu nanoparticles deposited on the surface.In Figure 4, the photo-acoustic spectra of the different structures are shown. These spectra have many distinct peaks at identical positions originated from the acoustic resonance in the experimental photo-acoustic cell. Apart from these resonance peaks, we focused our attention on the relative amplitudes of the obtained spectra. The photo-acoustic response of crystalline Si wafer increases when copper nanoparticles are deposited on the surface despite the absorption located at 785 nm is quite similar for both samples.

On the other hand, the photo-acoustic response of the nanoPS layer is about two times higher than that of c-Si. This increase is mainly due to the lower thermal conductivity and higher light absorption of the nanoPS layers. When Cu nanoparticles are deposited into nanoPS, the photo-acoustic response significantly increases, although the absorption at 785 nm is quite similar. This increase can be associated to the Cu nanoparticles acting as localized heat sources near the surface where the heat exchange between the Cu nanoparticles and air is more efficient, increasing the total temperature of the devices and therefore the photo-acoustic intensity.

**Figure 5:**
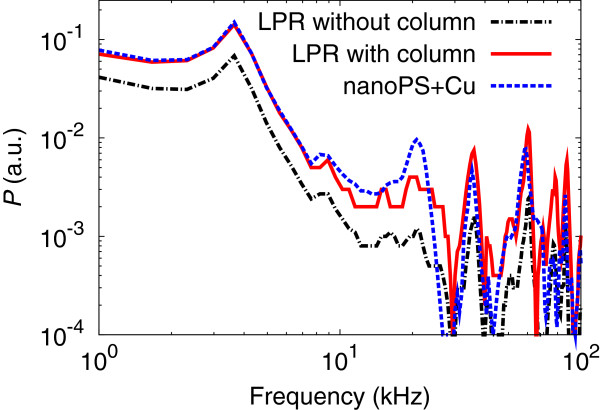
Photo-acoustic response from nanoPS + Cu and LPR with and without a porous column layer.

Aiming at evaluating the enhancement of the photo-acoustic response, the photo-acoustic spectra of nanoPS + Cu (*A* = 0.94) and local plasmon resonators (LPR) (*A* = 0.93 ~ 0.95) were compared, all of which have high optical absorption, *A*, at the wavelength of 785 nm (Figure 5). The local plasmon resonators have Au NPs/dielectric/Ag mirror structures that can be prepared by using the dynamic oblique angle deposition technique [13]. Due to the strong interference, their light absorption is highly localized in the Au NP layer, which enhances the periodic temperature change of the sample surface and simultaneously contributes to emit strong photo-acoustic response, compared to the bulk absorber such as graphite [19]. It has been shown that the local plasmon resonator whose dielectric layer consists of porous SiO_2_ column layers (Figure 5, LPR with columns) generates two to approximately five times larger photo-acoustic response than that whose dielectric layer consists of a solid SiO_2_ layer (Figure 5, LPR without columns) [13], while the photo-acoustic response from nanoPS + Cu is almost the same as that from the local plasmon resonator. This enhancement is in terms of the low thermal conductivity of the porous column layer and nanoPS layer, which contributes to the reduction of the amount of heat escaping to the substrate and to the efficient photo-acoustic emission from metal nanoparticle arrays.

**Figure 6:**
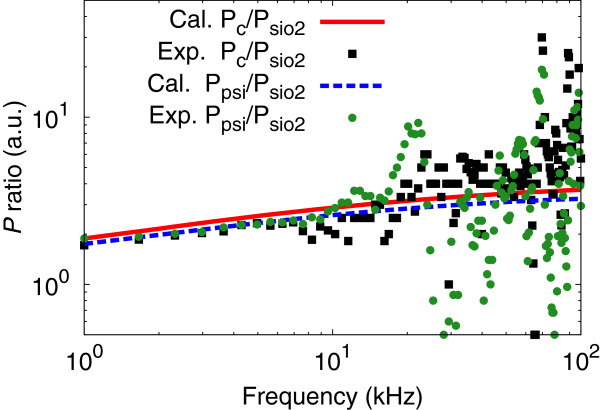
**Ratio of photo-acoustic responses *P***_**c**_**/*P***_**sio2 **_**(black squares) and *P***_**psi**_**/*P***_**sio2 **_**(green circles) as a function of laser modulation frequency.***P*_c_, *P*_sio2_, and *P*_psi_ represent the photo-acoustic response of the local plasmon resonator with and without a porous column layer and that of nanoPS + Cu, respectively. The red and blue dashed lines show the calculation result for *P*_c_/*P*_sio2_ and *P*_psi_/*P*_sio2_, respectively.

In order to verify the contribution of the porous column and nanoPS layers to the efficient photo-acoustic emission, numerical analyses based on the one-dimensional heat transfer model were performed [13,20]. As already reported in [13], the enhancement ratio, *P*_c_/*P*_sio2_, where *P*_c_ and *P*_sio2_ represent the photo-acoustic response of LPR with and without a porous column layer, respectively, agrees well with the experimental results (black squares in Figure 6). This suggests that the photo-acoustic enhancement has its origin in the existence of the porous column layer with the low thermal conductivity.

On the other hand, the thermal conductivity of the nanoPS layer under Cu NPs (approximately 0.6 [W m^-1^ K^-1^]) [21] is in the same order of magnitude as that of the porous column layer (approximately 0.2 [W m^-1^ K^-1^]) [20] and expected to be a thermal insulation layer as the porous column layer. In fact, the calculated *P*_psi_/*P*_sio2_, where *P*_psi_ represents the photo-acoustic amplitude of nanoPS + Cu, agrees well with the experimental results, suggesting that the strong photo-acoustic emission from nanoPS + Cu can be well understood by the low thermal conductivity of the PSi layer. Consequently, we can conclude that hybrid systems composed of nanoPS and noble metal nanoparticles can be used for thermoplasmonic applications, having much simpler structures than the local plasmon resonator and can be fabricated without a vacuum system.

## Conclusions

In this report, the photo-assisted electroless deposition of copper on crystalline silicon and nanostructured porous silicon has been studied. This method has been demonstrated as an efficient technique to deposit copper nanoparticles not only on the surface but also inside the porous structures. The photo-acoustic response of the resulted devices has been measured showing a notable increase in the intensity when copper nanoparticles are deposited into nanostructured porous silicon. Accordingly, nanostructured copper/porous silicon hybrid systems have been shown to be very promising candidates for several applications in the field of thermoplasmonics, including the development of efficient sound-emitting devices.

## Competing interests

The authors declare that they have no competing interests.

## Authors’ contributions

GRS fabricated the samples, carried out the morphological and optical characterization, and drafted the manuscript. KN participated in the design of the study and carried out the photo-acoustic experiments. MS and RJMP conceived of the study, participated in its design and coordination, and helped to draft the manuscript. All authors read and approved the final manuscript.
